# Dietary management of inflammatory diseases

**DOI:** 10.1186/1479-5876-10-S3-P12

**Published:** 2012-11-28

**Authors:** Umar Bacha, Muhammad A Ali, Abdul Basit, Uzma Litaf

**Affiliations:** 1 Dept. of Food Science & Human Nutrition, University of Veterinary & animal Sciences, Lahore, Pakistan; 2 Kohat Institute of Medical Sciences, Kohat, Pakistan; 3 Dept. of Food Science & Technology, Agricultural University Peshawar, Pakistan

## 

Ulcerative colitis and Crohn’s disease are inflammatory diseases which are considered to be either associated with the genetic make-up of the individual or due to defective epithelial barrier. In the latter case, environment becomes favorable for proliferation of microorganisms [1] which then causes inflammation. The small intestine expresses gut-associated lymphoid tissue (GALT) to encounter pathogen [2] which (lymph nodes and payer’s patches etc.,) changes with changing in colonization of microbes [3]. There are trillion of bacteria on the human gut and this microbiota population depends on nutritional status of the subjects. A healthy nutrition thus promote healthy microflora and vice versa. In some stressful condition (chemotherapy or poor nutrition) the balance in microbiota disturbed. This leads to invasive microbes to invade the gut and intestine. Intestinal cells possess conserve detecting system of bacterial antigens. This leads to progression and regulation of innate and adaptive immune responses. The GALT cells (macrophages, dendritic cells) sense signal from molecules, MHCI, II & TLRs [4]. It would be imperative to characterize diet that promotes growth of microbes which expresses lipopolysaccharides, peptidoglycan etc., because microbes with the mentioned antigens causes inflammation.
